# Clinical significance of high *c-MYC* and low *MYCBP2* expression and their association with *Ikaros* dysfunction in adult acute lymphoblastic leukemia

**DOI:** 10.18632/oncotarget.5982

**Published:** 2015-10-17

**Authors:** Zheng Ge, Xing Guo, Jianyong Li, Melanie Hartman, Yuka Imamura Kawasawa, Sinisa Dovat, Chunhua Song

**Affiliations:** ^1^ The First Affiliated Hospital of Nanjing Medical University, Jiangsu Province Hospital, Department of Hematology, Nanjing 210029, China; ^2^ Pennsylvania State University College of Medicine, Department of Pediatrics, Hershey, PA 17033, USA; ^3^ Institute for Personalized Medicine, Departments of Biochemistry and Molecular Biology and Pharmacology, Penn State College of Medicine, Hershey, PA 17033, USA

**Keywords:** c-MYC, MYCBP2, Ikaros, adult leukemia, ALL

## Abstract

Increased expression of *c-MYC* is observed in both Acute Myeloid Leukemia (AML) and T- cell Acute Lymphoblastic Leukemia (T-ALL). MYC binding protein 2 (MYCBP2) is a probable E3 ubiquitin ligase and its function in leukemia is unknown. *IKZF1* deletion is associated with the development and poor outcome of ALL. Here, we observed significant high *c-MYC* expression and low *MYCBP2* expression in adult ALL patients. Patients with high *c-MYC* expression and/or low *MYCBP2* expression had higher WBC counts and a higher percentage of CD34+ or CD33+ cells, as well as splenomegaly, liver infiltration, higher BM blasts, and lower CR rate. *Ikaros* bound to the regulatory regions of *c-MYC* and *MYCBP2*, suppressed *c-MYC* and increased *MYCBP2* expression in ALL cells. Expression of *c-MYC* mRNA was significantly higher in patients with *IKZF1* deletion; conversely *MYCBP2* mRNA expression was significantly lower in those patients. A CK2 inhibitor, which acts as an Ikaros activator, also suppressed *c-MYC* and increased *MYCBP2* expression in an Ikaros (*IKZF1*) dependent manner in the ALL cells. In summary, our data indicated the correlation of high *c-MYC* expression, low *MYCBP2* expression and high *c-MYC* plus low *MYCBP2* expression with high-risk factors and proliferation markers in adult ALL patients. Our data also revealed an oncogenic role for an *Ikaros*/*MYCBP2*/*c-MYC* axis in adult ALL, providing a mechanism of target therapies that activate Ikaros in adult ALL.

## INTRODUCTION

The MYC families of proteins are transcription factors with essential roles in cell growth and proliferation through their ability to regulate gene expression [1, 2]. Activation or amplification of the MYC oncogene family is one of the most frequent events associated with cancer [2]. *c-MYC* is frequently activated in acute myeloid leukemia (AML), and plays an important role in the induction of leukemogenesis [3, 4]. High *c-MYC* expression, a result of activating mutations in the *Flt3* receptor tyrosine kinase, correlates with poor prognosis in AML [5, 6]. *c-MYC* is also frequently reported to be upregulated in acute lymphoblastic leukemia (ALL), however, the correlation of *c-MYC* expression with clinical features of ALL has not been fully described. The cause of *c-MYC* overexpression in adult ALL is also unknown.

*MYCBP2* is likely an E3 ubiquitin ligase that binds specifically to MYC [7]. The region in *MYC,* which is responsible for MYCBP2 interaction, is frequently mutated in Burkitt's and AIDS-associated lymphomas, indicating *MYCBP2* suppress *MYC* activity [7]. Moreover, recently it is found that MiR-1247-5p overexpression resulted in a decreased expression of *MYCBP2* in prostate cancer [8]. Additionally, it is reported that *MYCBP2* is a candidate for the transformation-associated gene that maps to the 13q22.3 locus in Angioimmunoblastic T-cell lymphoma (AITL) [9]. However both the expression of *MYCBP2* and its correlation with clinical features are unknown in ALL.

*IKZF1* (*Ikaros*) encodes a kruppel-like zinc finger protein that is essential for normal hematopoiesis and acts as a tumor suppressor in ALL. The impairment of *Ikaros* function, as a result of deletion and/or inactivating mutation of a single *IKZF1* allele, is linked to the development of ALL that is characterized by a high rate of relapse and poor outcome. Therefore, *Ikaros* inactivation results in high-risk leukemia that is resistant to treatment. *Ikaros* exerts its anti-tumor effect by regulation of its target genes. *Ikaros* activates or represses expression of target genes by directly recruiting the NuRD/Mi2 or SWI/SNF chromatin remodeling complexes [10]. CK2 directly phosphorylate Ikaros, which results in suppression of its activity [11–14]. Recently we found that CK2 inhibition restores Ikaros function in ALL cells (15, 16). CK2 inhibitors can be used as Ikaros activator (15, 16). We also identified Ikaros binding profiling in ALL cells (15), and found that Ikaros exert its antitumor effect by regulating the expression of its gene targets (15); and CK2 inhibitors restore Ikaros function by increasing Ikaros binding to the gene targets and regulation of their expression in ALL cells [15, 16].

Here, we observed the expression of *c-MYC* and *MYCBP2* and their correlation with clinical features in adult ALL. We found *c-MYC* expression is negatively correlated with *MYCBP2* expression in ALL; and high *c-MYC* expression and/or low *MYCBP2* expression is associated with high-risk leukemia. We also observed the obvious Ikaros binding peaks in promoter region of *c-MYC* and *MYCBP2* in ALL cells by ChIP-seq, and found that *Ikaros* directly suppresses *c-MYC* and activates *MYCBP2* expression. Our results suggest that *Ikaros* dysfunction is partially responsible for the changes of *c-MYC* and *MYCBP2* in adult ALL.

## RESULTS

### Association of *c-MYC* expression with characteristics of adult ALL

**Figure 1:**
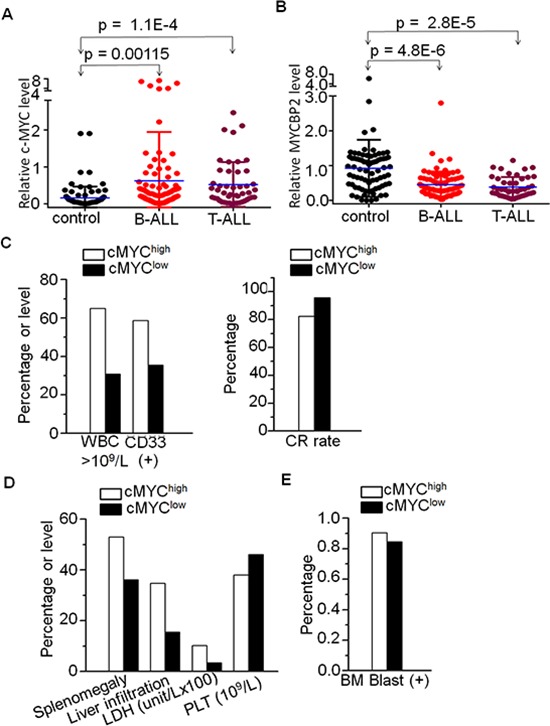
High *c-MYC* and low *MYCBP2* expression and correlation with clinical features in ALL patients **A.** Comparison of *c-MYC* expression in B-ALL and T-ALL with normal BM control; **B.** Comparison of *MYCBP2* expression in B-ALL and T-ALL with normal BM control; **C.** Correlation of high *c-MYC* expression with high risk factors (high WBC, CD13+ cells, and low CR rate); **D–E.** Correlation of high *c-MYC* expression with proliferation indicators.

We assessed *c-MYC* mRNA expression in 104 newly diagnosed adult B-ALL and 47 T-ALL patients. We found that *c-MYC* expression is significantly higher in both B-ALL and T-ALL patients compared to normal control (Fig. 1A). Patients were divided into high (*n* = 66) and low (*n* = 85) *c-MYC* expression groups. Patients with high *c-MYC* expression showed higher median white blood cell counts (WBC) (64.9 × 10^9^/L vs 30.9 × 10^9^/L, *P* = 0.009), a higher percentage of CD33(+) cells (58.7% vs 35.4%, *P* = 0.006), and a lower complete remission (CR) rate (82.3% vs 95.8%, *P* = 0.010) than those with low *c-MYC* expression (Supplemental Table 1, Fig. 1C). The percentage of patients exhibiting splenomegaly, liver infiltration, and increased lactate dehydrogenase (LDH) was significantly higher in the high *c-MYC* expression group than in the low expression group (53.0% vs 36.1%, *P* = 0.039; 34.8% vs 15.5%, *P* = 0.006; 49.2% vs 31.2%, *P* = 0.033) (Supplemental Table 1, Fig. 1D). The high *c-MYC* expression group had a significantly lower median PLT and high median BM blast than the low *c-MYC* expression group (38.0 vs 46.0, *P* = 0.035; 90.4% vs 84.4%, *P* = 0.006) (supplemental Table 1, Fig. 1D and 1E). No significant differences in *c-MYC* expression were observed with age, sex, or peripheral blood blasts. These data indicate that high *c-MYC* expression is associated with high-risk ALL.

### Association of *MYCBP2* expression with characteristics of adult ALL

We also assessed *MYCBP2* mRNA expression in 85 and 46 newly diagnosed adult B-ALL and T-ALL patients, respectively. We found that *MYCBP2* expression is significantly lower in both B-ALL and T-ALL patients when compared to normal control (Fig. 1B). Patients were divided into high (*n* = 65) and low (*n* = 66) *MYCBP2* expression groups. The high expression group showed lower median WBC counts (30.2 × 10^9^/L vs 46.5 × 10^9^/L, *P* = 0.025), a lower median percentage of CD33(+) cells (32.3% vs 57.1%, *P* = 0.004) and bone marrow blasts (87.2% vs 88.8%, *P* = 0.042) than the low expression group (Supplemental Table 2). The percentage of patients exhibiting splenomegaly and liver infiltration was significantly higher in the low *MYCBP2* expression group than in the high expression group (54.3% vs 35.9%, *P* = 0.033; 35.7% vs 15.4%, *P* = 0.007) (Supplemental Table 2). No significant differences in *MYCBP2* expression were observed with age, sex, or CR rate. These data indicated that low *MYCBP2* expression is to some extent associated with high-risk ALL.

### Association of *c-MYC*^high^*MYCBP2*^low^ expression with characteristics of adult ALL

**Figure 2:**
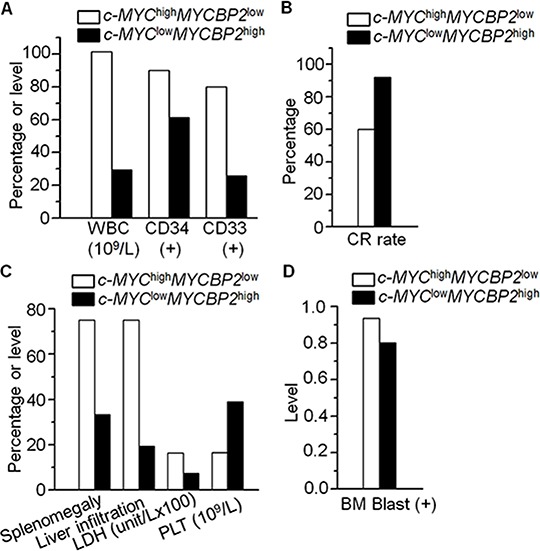
Correlation of *c-MYC*^high^ *MYCBP2*^low^ expression with clinical features in ALL **A–B.** Comparison of high risk factors (high WBC, CD34+, CD33+) (A) and low CR rate (B) in patients with *c-MYC*^high^ *MYCBP2*^low^ expression with those in patients with *c-MYC*^low^ *MYCBP2*^high^ expression; **C–D.** Comparison of percentage or level of splenomegaly, liver infiltration, LDH and PLT (C) and BM blast (D) in these two patients' groups.

We also observed that *c-MYC* mRNA expression is negatively correlated with *MYCBP2* expression in the ALL patients (Supplemental Fig. 1A). We analyzed the correlation of *c-MYC* with *MYCBP2* in other reported cohorts [16, 17] and found that *c-MYC* expression is negatively correlated with *MYCBP2* expression (Supplemental Fig. 1B). We also observed the c-MYC is interacts with MYCBP2 in ALL cells (Supplemental Fig. 2). We further compared the clinical features of patients with both high *c-MYC* expression and low *MYCBP2* expression (c-MYC^high^*MYCBP2*^low^) with that of patients with both low *c-MYC* expression and high *MYCBP2* expression (*c-MYC*^low^*MYCBP2*^high^). Our data showed that c-MYC^high^*MYCBP2*^low^ patients showed higher median WBC counts (101.5 × 10^9^/L vs 29.4 × 10^9^/L, *P* = 0.007), a higher percentage of CD34(+) and CD33(+) cells (90.0% vs 61.3%, *P* = 0.025; 80.0% vs 25.8%, *P* = 0.000), and a lower CR rate (60.0% vs 92.0%, *P* = 0.027) than *c-MYC*^low^*MYCBP2*^high^ patients (supplemental Table 3, Fig. 2A and 2B). The percentage of patients exhibiting splenomegaly, liver infiltration, increased LDH (>1000 u/L) and BM blasts was significantly higher in the *c-MYC*^high^*MYCBP2*^low^ group than in the *c-MYC*^low^*MYCBP2*^high^ group (75.0% vs 33.3%, *P* = 0.004; 75.0% vs 19.4%, *P* = 0.000; 70.0% vs 29.0%, *P* = 0.004, 93.6% vs 80.2%, *P* = 0.001) (supplemental Table 3, Fig. 2C and 2D). The *c-MYC*^high^*MYCBP2*^low^ group had a significantly lower *median PLT* than the *c-MYC*^low^*MYCBP2*^high^ group (16.5% vs 39.0%, *P* = 0.013) (Supplemental Table 3, Fig. 2C). No significant differences between the two groups were observed with age, sex, or peripheral blood blasts. These data indicate that *c-MYC* high expression with *MYCBP2* low expression is correlated with high-risk ALL.

### *Ikaros* binds to the promoter of *c-MYC* and *MYCBP2* and regulate their expression

**Figure 3:**
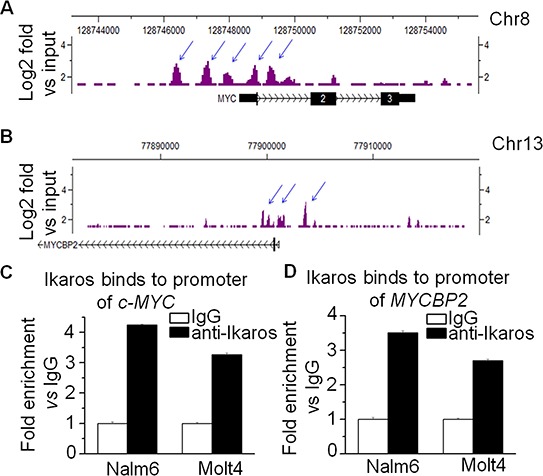
*Ikaros* binds the promoters of *c-MYC* and *MYCBP2* **A–B.** *Ikaros* binding peaks on the promoter of c-MYC (A) and of MYCBP2 (B) **C–D.** Ikaros binds to the promoter of *c-MYC* (C) and *MYCBP2* (D) in Nalm6 B-ALL and Molt4 T-ALL cells by qChIP assay.

**Figure 4:**
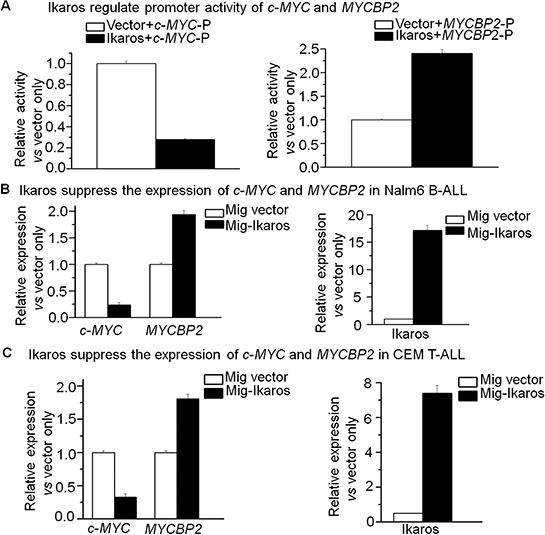
*Ikaros* suppresses the transcription of *c-MYC* and *MYCBP2* **A.** The promoter activity of *c-MYC* and *MYCBP2* promoters by luciferase reporter assay following transfection with *Ikaros* or control vector in HEK293 cells; *C-MYC*-p: *c-MYC* promoter; *MYCBP2*-p: *MYCBP2* promoter; **B–C.** Expression of *Ikaros* target genes (*c-MYC* and *MYCBP2*) in Nalm6 cells (B) and CEM T-ALL cells (C) transduced with vector containing *Ikaros* as compared to control vector.

**Figure 5:**
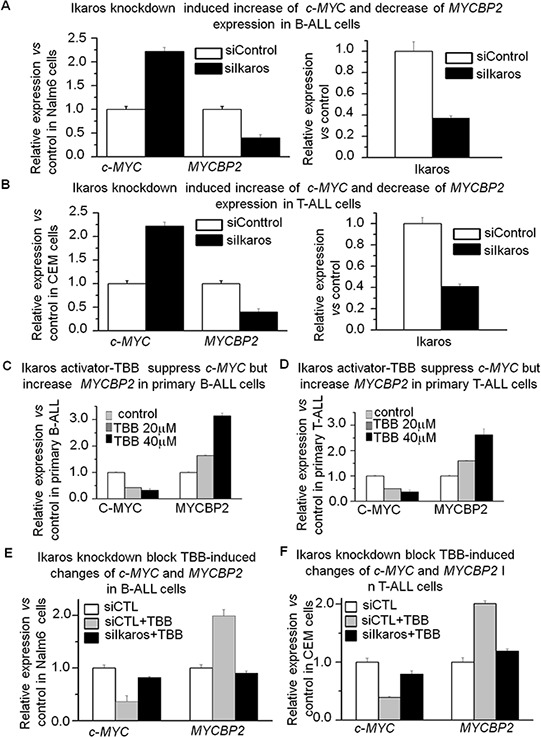
*Ikaros* knockdown induces upregulation of *c-MYC* and downregulation of *MYCBP2* **A–B.** qRT-PCR of *c-MYC*, *MYCBP2*, and *Ikaros* expression in Nalm6 cells (A) and CEM cells (B) following *Ikaros* shRNA treatment as compared to scramble shRNA cells. Gene expression is determined by RT-qPCR using total RNA isolated from the cells transfected with scramble shRNA (siControl) or *Ikaros* shRNA (si*Ikaros*) for 2 days; **C.** Effect of Ikaros activator (CK2 inhibitor, TBB) on expression of *c-MYC* and *MYCBP2* in primary B-ALL (C) and primary T-ALL **D.** cells with TBB treatment for 2 days; **E–F.** Ikaros knockdown rescues the TBB-induced change of *c-MYC* and *MYCBP2* in Nalm6 (E) and CEM (F) cells.

In order to understand how *c-MYC* and *MYCBNP2* expression are regulated in ALL, we analyzed the transcription factor motifs in the promoter region of *c-MYC* and *MYCBP2*. We identified strong *Ikaros* binding motifs in the promoter region of each gene. *c-MYC* is reported to be a direct target of *Ikaros*. Our *Ikaros* ChIP-seq data also showed strong *Ikaros* binding peaks in the promoter region of both *c-MYC* and *MYCBP2* (Fig. 3A and 3B). We explored the binding of *Ikaros* on the promoter regions of *c-MYC* and *MYCBP2* by qChIP and found that *Ikaros* significantly binds to their promoter regions in Nalm6 B-ALL and Molt4 T-ALL cells (Fig. 3C and 3D), and CEM T-ALL cells (data not show). We further observed that *Ikaros* suppresses the promoter activity of *c-MYC* and activates that of *MYCBP2* by luciferase reporter assay (Fig. 4A); and Ikaros knockdown block Ikaros-induced effect on the promoter activity of c-MYC and MYCBP2 (Supplemental Fig. 3). These data indicated that direct effect of Ikaros on transcription of *c-MYC* and *MYCBP2*. Moreover, expression of *Ikaros* suppressed *c-MYC* mRNA level and increased *MYCBP2* mRNA level in both Nalm6 and CEM cells (Fig. 4B and 4C), and this effect was observed in protein level (Supplemental Fig. 4A). Conversely, *Ikaros* knockdown induced an increase in *c-MYC* expression and a decrease in *MYCBP2* expression in Nalm6 (Fig. 5A) and CEM cells (Fig. 5B). The efficient knockdown for Ikaros was evidenced in mRNA level (Right panel for Fig. 5A and 5B) and protein level (Supplemental Fig. 4B). Treatment of Nalm6 and CEM cells with Ikaros activator, TBB (CK2 inhibitor) could suppress *c-MYC* expression and increase *MYCBP2* expression in a dose-dependent manner (Fig. 5C and 5D). Ikaros knockdown with shRNA could block the TBB-induced decrease in *c-MYC* expression and increase in *MYCBP2* expression (Fig. 5E and 5F). These data indicate that both *c-MYC* and *MYCBP2* are direct *Ikaros* targets in ALL and *Ikaros* regulates their expression.

### *c-MYC* and *MYCBP2* expression in patients with an *Ikaros* deletion

We explored *Ikaros* mRNA level by qPCR in our cohort study, and analyzed the correlation of Ikaros expression with clinical feature in the patients (Supplemental Table 4). We found that patients with low *Ikaros* expression showed higher median WBC counts (64.9 × 10^9^/L vs 29.5 × 10^9^/L, *P* = 0.016), higher percentage of CD13(+) cells (57.1% vs 33.3%, *P* = 0.009) and CD33 (+) cells (60.0% vs 40.5%, *P* = 0.033), higher percentage of liver infiltration and splenomegaly (41.5% vs 11.4%, *P* = 0.000; 56.6% vs 34.6%, *P* = 0.013) and lower CR rate (79.2% vs 94.3%, *P* = 0.013) than those with high *Ikaros* expression. These data indicated that low *Ikaros* expression, as that of high *c-MYC* and low *MYCBP2* is correlated with high-risk ALL.

**Figure 6:**
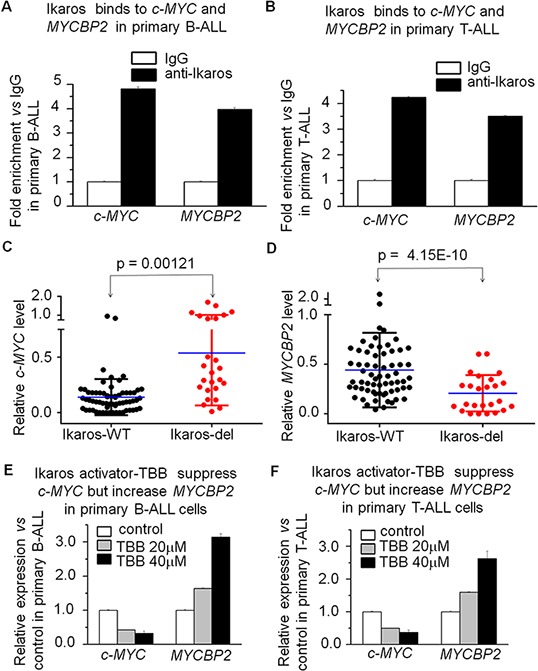
*Ikaros* binds to the *c-MYC* and *MYCBP2* promoters and *Ikaros* deletion results in changes of their expression in primary ALL cells **A–B.** *Ikaros* binding to the promoters of *c-MYC* and *MYCBP2* in primary B-ALL (A) and T-ALL (B) cells; **C–D.** comparison of *c-MYC* (C) and MYCBP2 (D) in patients with or without Ikaros deletion; **E–F.** Effect of Ikaros activator (CK2 inhibitor, TBB) on expression of *c-MYC* and *MYCBP2* in primary B-ALL(E) and T-ALL (F) cells with TBB treatment for 2 days.

In order to further explore the relationship of *Ikaros* with *c-MYC* and *MYCBP2* expression, we observed *Ikaros* binding to their promoters in primary B-ALL and T-ALL cells (Fig. 6A and 6B). We analyzed the correlation of *Ikaros* expression with that of *c-MYC* or *MYCBP2* in the reported cohort of B-ALL or T-ALL patients [17–19], and found that *Ikaros* expression is negatively correlated with high *c-MYC* expression and low *MYCBP2* expression in both B-ALL (Supplemental Fig. 5A and 5B) and T-ALL (Supplemental Fig. 6A and 6B). We also analyzed the correlation of *Ikaros* mRNA level with that of *c-MYC* and *MYCBP2* in our cohort study, and found that Ikaros is negatively correlated with *c-MYC* expression and positively correlated with *MYCBP2* expression (Supplemental Fig. 7A and 7B). Importantly, *c-MYC* expression was significantly increased and *MYCBP2* expression significantly decreased in patients with *Ikaros* deletion compared to that of *Ikaros* wild type (Fig. 6C and 6D). These data further demonstrated a regulatory effect of *Ikaros* on both *c-MYC* and *MYCBP2* in ALL patients and suggested that *Ikaros* deletion is one of the reasons for high *c-MYC* and low *MYCBP2* expression in the patients. Additionally, TBB can suppress *c-MYC* expression and increase *MYCBP2* expression in primary B-ALL (Fig. 6E) and T-ALL (Fig. 6F). This data not only indicated the effect of Ikaros activator on their expression, but also suggested that Ikaros-induced changes of *c-MYC/MYCBP2* expression is at least partially responsible for the success of CK2 inhibitors in ALL therapy.

## DISCUSSION

Our findings indicate the significance of high *c-MYC*, low *MYCBP2* and low Ikaros expression in ALL patients, also demonstrate *c-MYC* and *MYCBP2* is the direct target of *Ikaros* and reveal a model for the oncogenic effect of an *Ikaros*/*MYCBP2*/*c-MYC* axis in adult ALL. *Ikaros* dysfunction results in the upregulation of *c-MYC* and downregulation of *MYCBP2*. This is the first report shows that *Ikaros* dysfunction results in the increase of *c-MYC* and decrease of *MYCBP2,* as well as a negative correlation between *c-MYC* and *MYCBP2* in ALL patients.

*MYC*, as one of the key transcription factors in hematopoiesis, is frequently overexpressed in human acute lymphoblastic and myeloid leukemia [20–27]. We observed a significant increase in *c-MYC* mRNA in adult B-ALL and T-ALL compared to that in normal bone marrow. We also observed that high *c-MYC* expression is correlated with proliferation markers such as splenomegaly, liver infiltration, high LDH and BM blasts, and high-risk ALL factors: high WBC, CD33+ cells, and low CR rates. These findings indicate the oncogenic effect of high *c-MYC* expression.

The expression of *MYCBP2* in ALL is not well determined. Here, we observed a significant decreased *MYCBP2* expression in B-ALL and T-ALL patients. Low *MYCBP2* expression is correlated with both proliferation markers and high-risk factors for ALL. We also observed a negative correlation between *c-MYC* and *MYCBP2* expression. More importantly, *c-MYC*^high^*MYCBP2*^low^ patients showed higher median WBC counts, higher percentage of CD34(+) and CD33(+) cells, higher rate of splenomegaly, liver infiltration, increased LDH, and lower CR rate compared with that of c-MYC^low^MYCBP2^high^ patients. These data indicate that high *c-MYC* and low *MYCBP2* expression have a synergistic oncogenic effect in ALL patients.

In human ALL, upregulation of *c-MYC* has been reported through chromosome translocations [28], aberrant *c-MYC* stability [29], and genetic gene fusion [6, 30]. We also reported that high *LEF1* expression increases *c-MYC* expression in ALL [31]. *Ikaros* is reported to inhibit pre-B cell proliferation by suppression of *c-MYC* expression [32]. Here, we found *c-MYC* expression is significantly increased in patients with an *Ikaros* deletion; and *c-MYC* expression is negatively correlated with *Ikaros* expression in ALL patients in the cohort studies. These data indicated that *Ikaros* plays an important role in the suppression of *c-MYC* expression in ALL, and that *Ikaros* deletion is one of the major reasons for high *c-MYC* expression in ALL patients. Moreover, *Ikaros* also induces *MYCBP2* expression. *Ikaros* expression is positively correlated with *MYCBP2* expression in ours and the other reported cohort studies. We also observed that *MYCBP2* expression is significantly increased in patients with an *Ikaros* deletion. These data indicated that *Ikaros* dysfunction is an important mechanism underlying low *MYCBP2* expression in the patients.

Our results also showed that Ikaros binds to *c-MYC and MYCBP2* promoter, directly suppresses or increase their promoter activity, and further results in the changes of their expression in ALL cells. As a transcription factor, Ikaros regulates gene expression by recruiting the NuRD/Mi-2 or SWI/SNF chromatin remodeling complexes [10, 15, 37]. Whether *Ikaros* regulating *c-MYC* and *MYCBP2* expression through chromatin remodeling needs to be further clarified.

CK2 inhibitor, TBB as an indirect Ikaros activator, inhibits *c-MYC* expression and promotes *MYCBP2* expression in an Ikaros-dependent manner. CK2 inhibitors are reported to be potential therapies for leukemia [15, 13, 33]. Therefore, our data suggests that CK2 inhibitors exert their anti-leukemia effect through Ikaros-mediated suppression of *c-MYC* expression and activation of *MYCBP2* expression.

Interestingly, we observed a negative correlation between *c-MYC* and *MYCBP2* expression. We also found that *c-MYC* expression is negatively correlated with *MYCBP2* expression in microarray data of ALL patients from other cohort studies. MYCBP2 is a large protein that binds specifically to MYC [7]. The mechanism underlying the negative correlation needs to be clarified further. However, *MYCBP2* as an ubiquitin ligase may directly downregulate *c-MYC* protein. We did observe c-MYC is associated with MYCBP2 in ALL cells. *Ikaros*-induced low *MYCBP2* expression might further increase the c-MYC protein level in the patients.

In conclusion, we observed that *c-MYC* expression is significantly increased and *MYCBP2* expression significantly decreased in adult ALL patients. High *c-MYC* and/or low *MYCBP2* or low *Ikaros* expression is correlated with high-risk leukemia. *Ikaros* dysfunction is one of the underlying reasons for high *c-MYC* and low *MYCBP2* expression in ALL patients. Our data reveals the oncogenic effect of an *Ikaros*/*MYCBP2*/*c-MYC* axis in adult ALL, and suggests a mechanism by which CK2 inhibitors exert their anti-leukemia effect.

## MATERIALS AND METHODS

### Patients and samples

BM samples [90 male, 61 female; median age 32 (14–77) years old] with ALL (104 B-ALL, 47 T-ALL) were collected from 151 patients between June 2008 and June 2014 at the First Affiliated Hospital of Nanjing Medical University. The ALL diagnosis was made according to the cytogenetic, morphologic, immunophenotypic, and molecular criteria of WHO Diagnosis and Classification of ALL (2008). Written informed consent was provided before enrollment in the study by all patients in accordance with the Declaration of Helsinki. The cohort study was also approved by the Institutional Review Board of the Nanjing Medical University.

### Cytogenetic and molecular analyses

Conventional cytogenetic analysis was performed at the time of diagnosis by using unstimulated short-term cultures according to the recommendations of the International System for Human Cytogenetic Nomenclature (ISCN). At least 20 bone marrow metaphase cells were analyzed for each sample.

Flow cytometry was performed on fresh pretreatment BM samples for immunophenotypic analyses. A cell-surface antigen was defined as positive when fluorescence intensity of at least 20% of cells exceeded fluorescence of negative control as previously described [31].

### Cell culture reagents, plasmid construction, and retroviral gene transfer

Nalm6 and MOLT4 cells were obtained from the American Type Culture Collection (ATCC, Manassas, VA) and cultured in RPMI 1640 medium (Cellgro) supplemented with 10% fetal bovine serum (Hyclone). HEK 293T cells were cultured in DMEM (Cellgro) supplemented with 10% fetal calf serum and 1% L-glutamine (Cellgro). Cells were incubated at 37°C in a humidified atmosphere of 5% CO_2_. Primary human B-ALL and T-ALL cells were cultured in RPMI 1640 medium (Cellgro) supplemented with 10% fetal bovine serum (Hyclone). Cells were cultured with or without TBB and collected for total RNA isolation. Human HA-tagged *Ikaros* (*IKZF1*) retroviral construct and retroviral production was described previously [11, 12, 34].

### Luciferase assay

The pGL3 luciferase reporter construct for the *c-MYC* promoter was previously reported [31] and the pGL3-*MYCBP2* promoter construct was purchased from Addgene. Transient luciferase assays were performed in HEK293T cells using Promega luciferase assay reagents and measured with a luminometer following the manufacturer's instructions. Luciferase activity was calculated as fold change relative to values obtained from pGL vector only control cells and expressed as a percentage of pcDNA 3.1-*Ikaros* transfection-induced luciferase activity versus that of pcDNA3.1 vector alone. All transfection and reporter assays were performed independently, in triplicate, at least three times.

For *Ikaros* knockdown, the same amount of *Ikaros* shRNA were transfected with the Ikaros and promoter constructs, and the resulting promoter activity was compared to that with scramble shRNA control.

### Real time-PCR

Total RNA was isolated using the RNeasy Mini Kit (QIAGEN). A 1 μg aliquot of RNA was reverse transcribed using the SuperScript™ First-Strand Synthesis System for RT-PCR Kit (Invitrogen). qRT-PCR was performed with qSTAR SYBR Master Mix (OriGene) using a StepOne Plus real-time PCR system (Applied Biosystems). Each experiment was performed in triplicate.

*c-MYC* and *MYCBP2* expression in patient samples was quantitated similarly by the formula achieved with serial dilutions of their plasmids as template standards as previously reported [31]. Gene expression values of *c-MYC* or *MYCBP2* were achieved in each patient by a formula obtained with a scatter graph of the Ct values from the serial dilutions of template plasmid as previously reported [31, 35]. The expression level of *c-MYC* or *MYCBP2* was subsequently normalized to 18s RNA and expressed as gene expression value of *c-MYC* or *MYCBP2*/18s RNA.

All the patients were divided into high and low *c-MYC/MYCBP2* expression groups (Q3-4 vs Q1-2, respectively), which was determined by SPSS 17.0.

The qPCR for *c-MYC* and *MYCBP2* expression was performed in Nalm6 and Molt4 cells transfected with *Ikaros.* The results were normalized to those obtained with 18sRNA and presented as fold induction over vector controls. Primers: *18s RNA*, Sense: 5′-GTAACCCGTTGAACCCCATT-3′, Antisense: 5′-CCATCCAATCGGTAGTAGCG-3′; *c-MYC* Sense: 5′-AATGAAAAGGCCCCCAAGGTAGTTATCC-3′, Anti-sense: 5′-GTCGTTTCCGCAACAAGTCCTCTTC-3′; *MYCBP2* Sense: 5′-TCACAGTGCAAG AAGGATACCAAA-3′, Anti-sense: 5′-TGAAAGC CAGCATCGTTCTTAGTC-3′.

### Quantitative chromatin immunoprecipitation (qChIP)

qChIP assays were performed by incubation of the chromatin with antibodies against *Ikaros* and normal rabbit IgG (Abcam) as control [16]. Enrichment of the ChIP sample over input was evaluated by qPCR with three or more replicates, using specific primers in the promoter region of *c-MYC* (forward: 5′-AGGGGA AGGGAGGGGAAGGGAAAG-3′, reverse: 5′-CCCTTTCCTCCCCTCCCCTCCT-3′) and *MYCBP2* (forward: 5′-GGTTTTCTCTGCCTTAAACTCTGAA-3′, reverse: 5′-GCTGCCAACAGGAAGATTTACTG-3′). The relative concentration of the qPCR product was presented as the fold change of the level of DNA in *Ikaros* samples in comparison to controls.

### Ikaros shRNA knockdown

Nalm6 cells were transiently transfected with human *Ikaros* shRNA constructs in the GFP vector (pGFP-v-RS) (Origene) using the Neon Transfection System (Invitrogen). The 29-mer scrambled shRNA cassette in the pGFP-VRS vector was also used as a control. After transfection for 1 day, Nalm6 and CEM cells with transfection efficiency around 80% (green cells) and greater than 95% cell viability were further treated with 20 μM TBB or vehicle control (0.01% DMSO) for 2 days and harvested for total RNA isolation. Knockdown of *Ikaros* was confirmed by measurement of *Ikaros* mRNA level using qPCR. Primers: *IKZF1*-F:5′-ggcgcggtgctcctcct-3′, *IKZF1*-R: 5′-tccgacacgccctacgaca-3′

### Co-immunoprecipitation and western blot

Nuclear extracts from Nalm6 and CEM cells were prepared as previous reported [36]. Nuclear extracts were precleared with protein A-Sepharose beads. The precleared extracts were incubated with 5 μg of antibody to c-Myc or MYCBP2 or normal rabbit IgG control for 2 hours, and then added the pre-washed protein A-Sepharose beads at 4°C overnight. Immunoprecipitated complexes were extensively washed with lysis buffer and then boiled in SDS sample buffer for 10 minutes. The immunoprecipitation products were run on SDS-PAGE and transferred to the membrane.

For western blot, membranes were blocked with 5% nonfat dry milk at room temperature for 1 hour and then incubated overnight at 4°C with primary antibody (anti-c-MYC, 1:500, Sigma; anti-MYCBP2; 1:1000, Abcam). After being washed, the membrane was incubated with goat anti-rabbit IgG conjugated to horseradish peroxidase (1:3000) at room temperature for 2 hours. The blots were developed by the enhanced chemiluminescence technique (ECL Plus, Amersham, Arlington Heights, IL) according to the manufacturer's instructions.

### Statistical analysis

Patients were divided into high and low *c-MYC or MYCBP2* expression groups (Q3-4 vs Q1-2, respectively). For quantitative parameters, overall differences between the cohorts were evaluated using a Mann – Whitney *U*-test. For qualitative parameters, overall group differences were analyzed using a χ^2^ test. All statistical analyses were performed using the SPSS 17.0 and *P* < 0.05 was considered statistically significant.

The experimental data are shown as the mean value with bars representing the standard error of the mean (S.E.M.). Determinations of statistical significance were performed using a Student *t*-test for comparisons of two groups or using analysis of variance (ANOVA) for comparing multiple groups. The *P* < 0.05 was considered statistically significant.

## SUPPLEMENTARY FIGURES AND TABLES


