# Supplementary material for: Clinical significance of high c-MYC and low MYCBP2 expression and their association with Ikaros dysfunction in adult acute lymphoblastic leukemia
Source: Oncotarget. 2015 Oct 17;6(39):42300–11. doi: 10.18632/oncotarget.5982 (PMC4747226; doi:10.18632/oncotarget.5982)
Supplement: Supplementary file 1 [file oncotarget-06-42300-s001.pdf]

SUPPLEMENTARY FIGURES AND TABLES

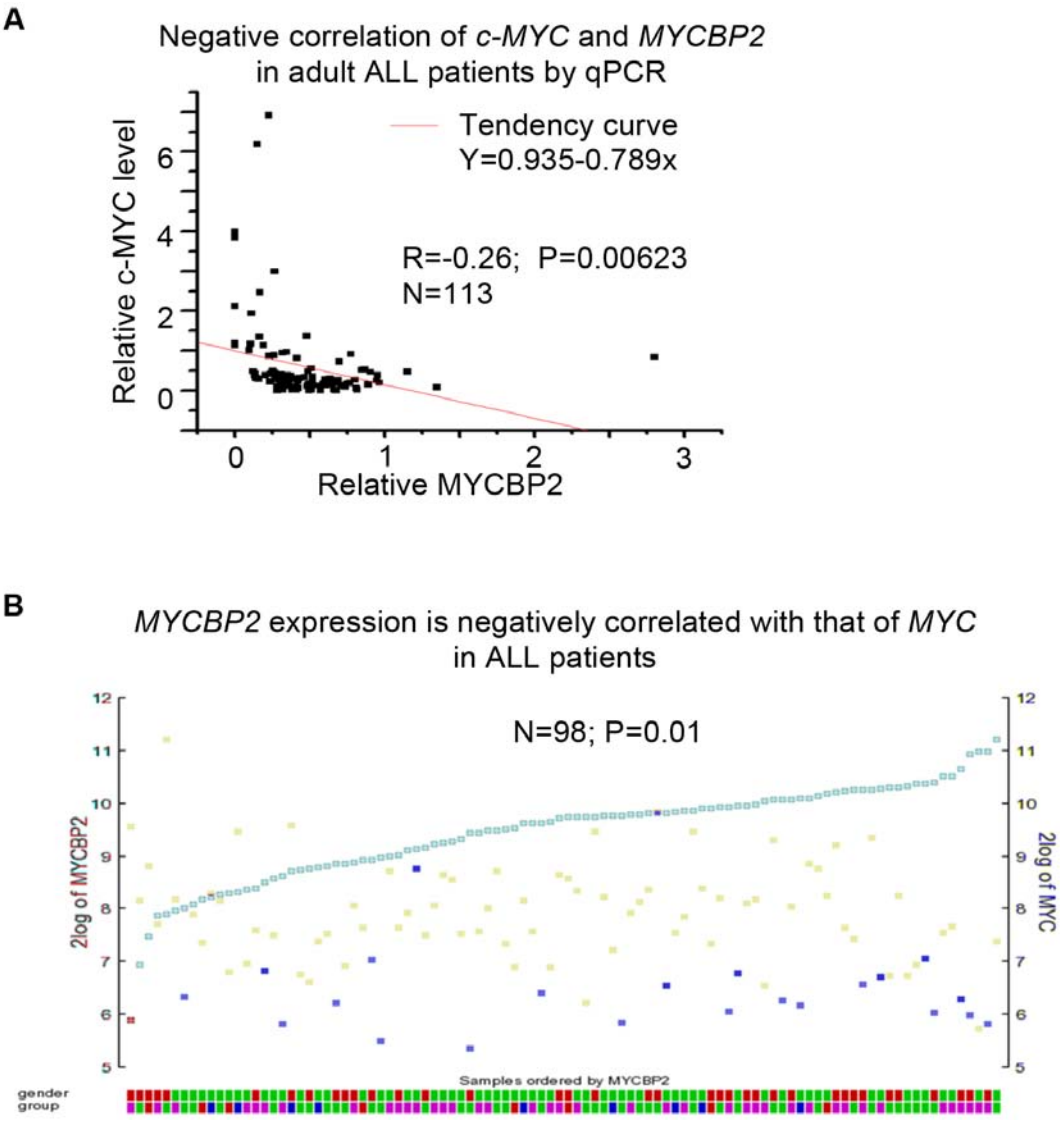

Supplementary Figure S1: Correlation of *MYC* expression with *MYCBP2* expression in the cohort of ALL patients. A. Correlation of *MYC* and *MYCBP2* expression identified by qPCR in the adult ALL cohort. **B.** Data generated from human oncogenomics sever with GEO database (GSE11877 and GSM267399). Pearson correlation is used to calculate the statistics. The $P < 0.05$ was considered statistically significant.

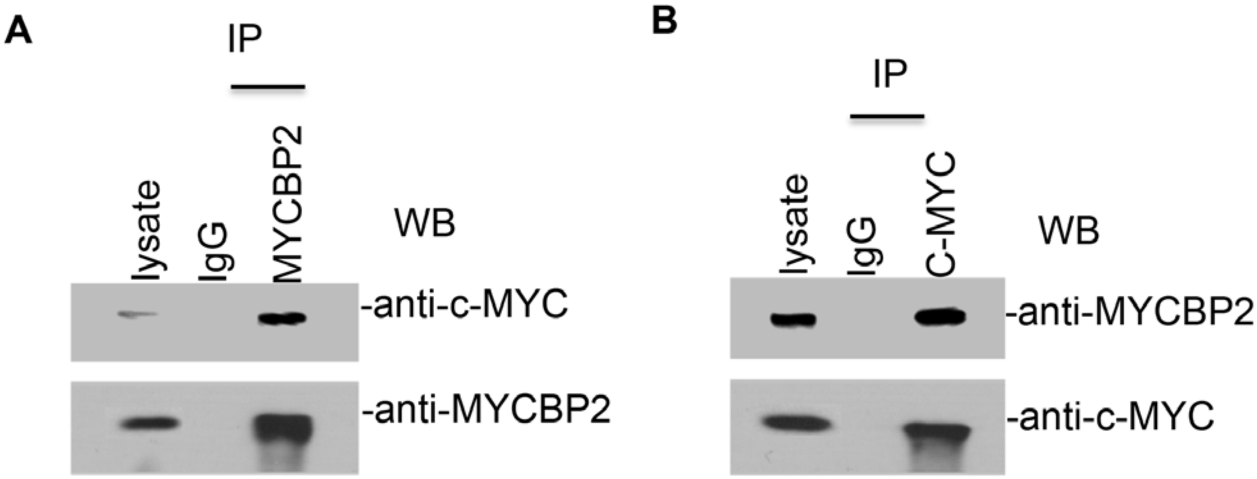

Supplementary Figure S2: *c-MYC* interacts with *MYCBP2* in Nalm6 cells. A. Co-immunoprecipitation of c-MYC by MYCBP2 in the cells; **B.** Co-immunoprecipitation of MYCBP2 by c-MYC in the cells. Total cell lysate was prepared in the cells with the lysis buffer as reported [15, 16, 36] and the co-immunoprecipitation was done as previous reported with anti-MYCBP2 or anti-c-MYC antibody, and with normal IgG as control. Co-immunoprecipitated c-MYC or MYCBP2 was detected by western blot.

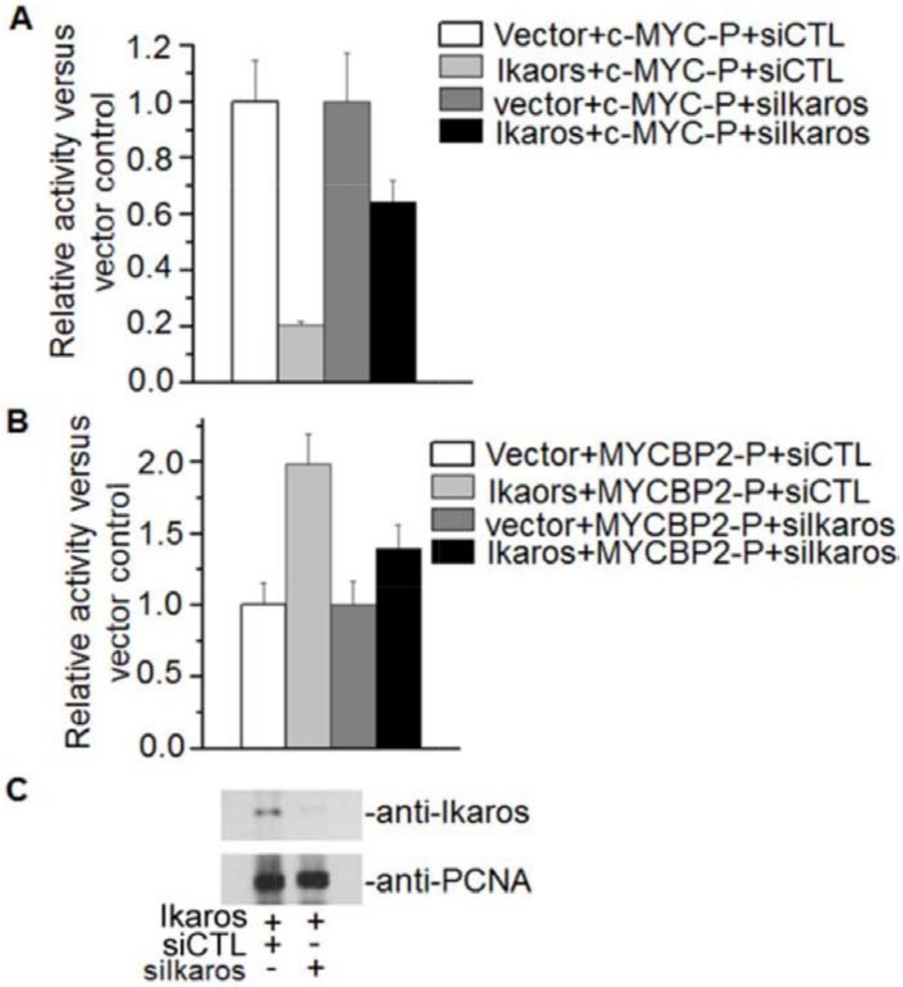

Supplementary Figure S3: Effect of *Ikaros* knockdown on *Ikaros* regulation of promoter activity. A. Ikaros knockdown block Ikaros-induced suppression of c-MYC promoter activity; Ikaros was transfected into the HEK293 cells with Ikaros shRNA (siIkaros) or scarmble shRNA (siCTL) as control . The luciferase reporter assay was done as described in the method part; **B.** Ikaros knockdown blocked the Ikaros-induced increase of MYCBP2 promoter activity; **C.** detection of Ikaros protein level in the Ikaros knockdown cells by Western blot as previous reported (15, 36).

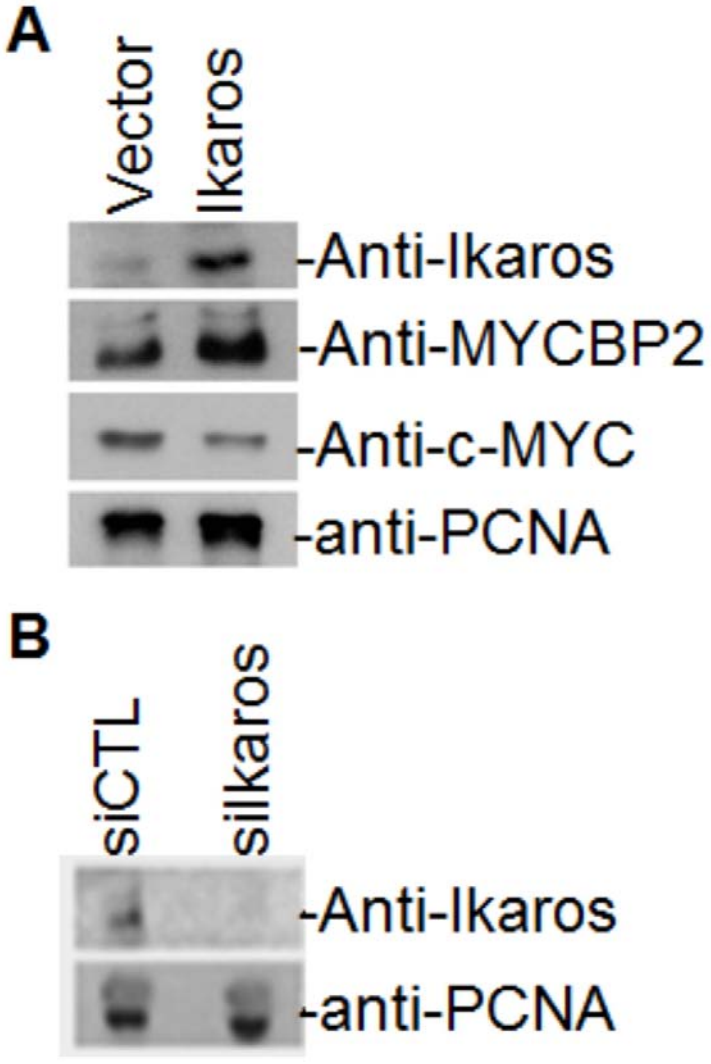

Supplementary Figure S4: Expression of *Ikaros* results in the expression change of *c-MYC* and *MYCBP2* in Nalm6 cells and *Ikaros* level in the knockdown cells. A. Expression of Ikaros results in the expression change of c-MYC and MYCBP2 in Nalm6 cells. Nuclear extracts from retroviral Ikaros transduced Nalm6 cells or vector only control is prepared as previous reported [36, 16]. The expression of the indicated genes is detected in the cells by western blot with indicated antibodies. **B.** Ikaros protein level was also detected by western blot in Nalm6 cells expressed with Ikaros shRNA (siIkaros) compared to that of scramble shRNA (siCTL).

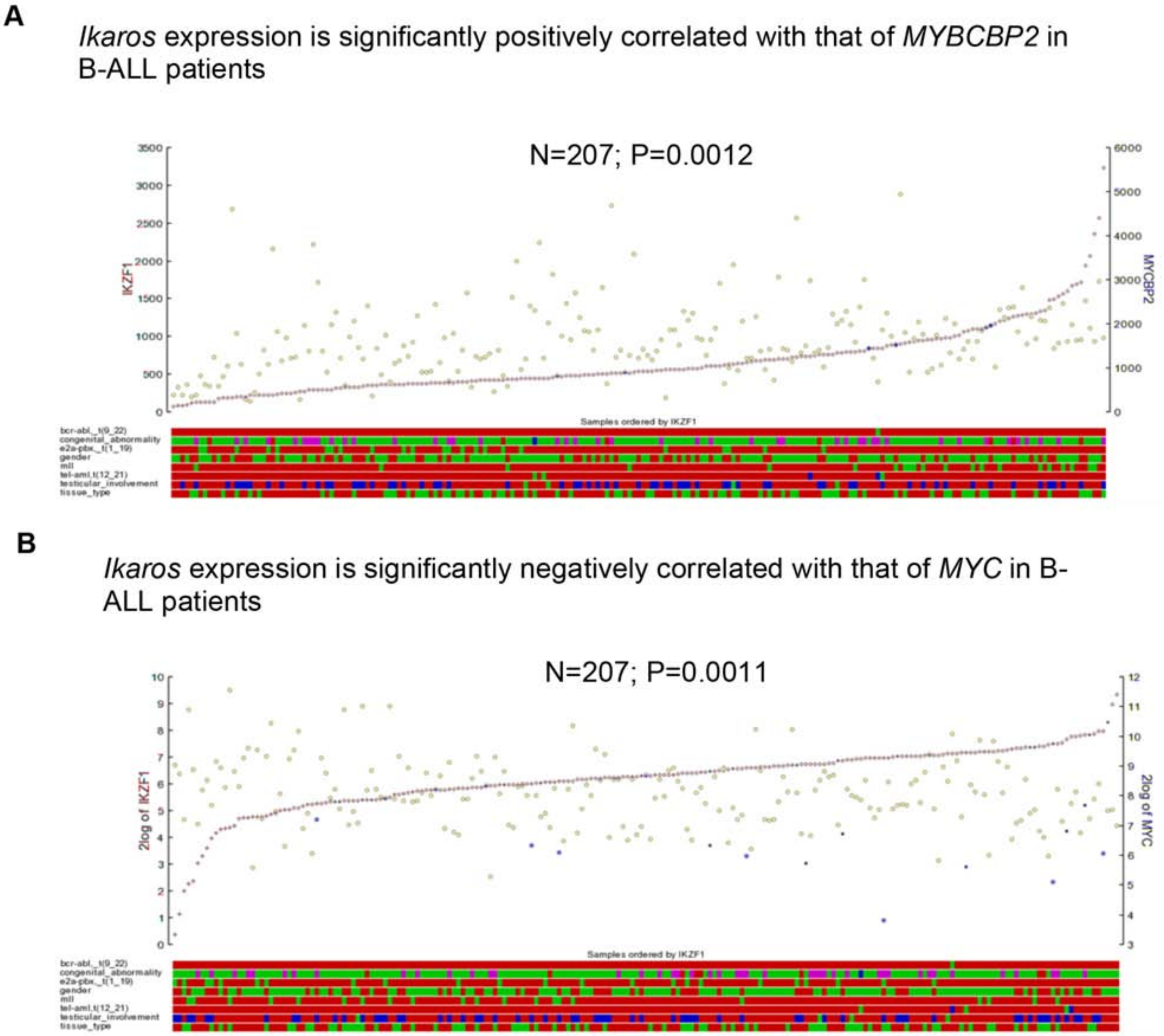

Supplementary Figure S5: Correlation of *MYC* and *MYCBP2* expression with *Ikaros* in the cohort of B-ALL patients. Data generated from human oncogenomics sever with GEO database (GSE11877). Pearson correlation is used to calculate the statistics. The $P < 0.05$ was considered statistically significant.

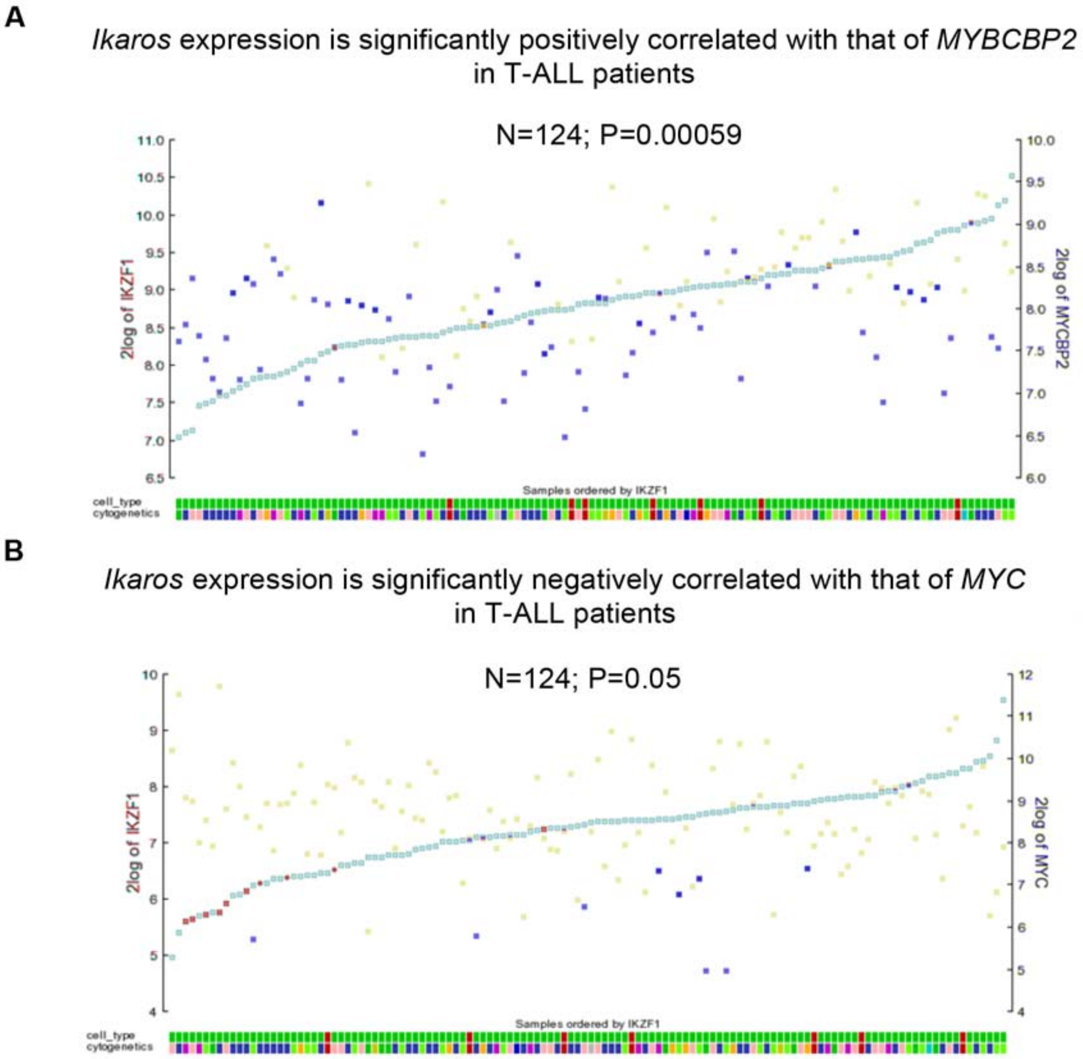

Supplementary Figure S6: Correlation of *MYC* and *MYCBP2* expression with *Ikaros* in the cohort of T-ALL patients. Data generated from human oncogenomics sever with GEO database (GSM267399). Pearson correlation is used to calculate the statistics. The $P < 0.05$ was considered statistically significant.

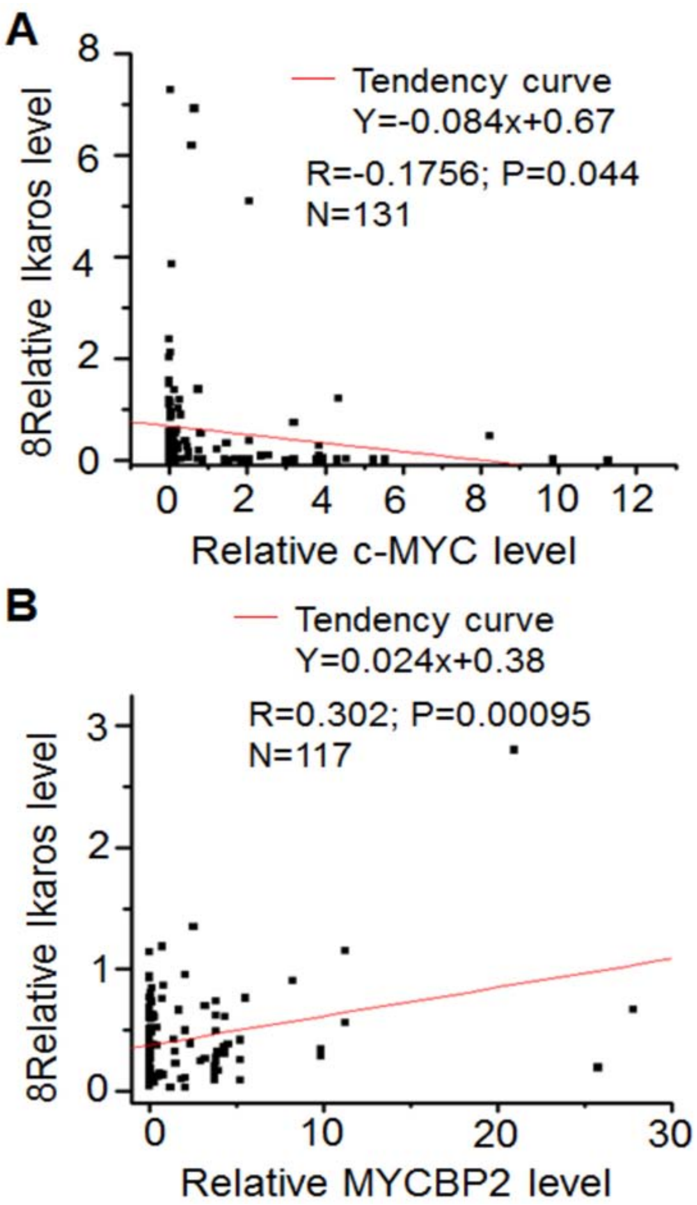

Supplementary Figure S7: Correlation of *Ikaros* mRNA level with that of *MYC* or *MYCBP2* in the ALL patients. A–B. The mRNA level of *Ikaros*, *c-MYC*, *MYCBP2* was detected by qPCR in ALL patients. Regression analysis of *Ikaros* level versus *c-MYC* level (A) and *MYCBP2* level (B).

Supplementary Table S1: The correlation of expression of *c-MYC* with clinical features in acute lymphoblastic leukemia

Characteristics	*c-MYC* high expression ($N = 66$)	*c-MYC* low expression ($N = 85$)	*P* value
Age (years)			
Median (range)	36.5 (14–77)	27 (14–75)	0.315
Sex (%)			
male	62.1	57.6	0.578
WBC, × 10^9/L			
median (range)	64.9 (0.9–487.9)	30.9 (2.0–626.0)	0.009
HGB,g/L			
median (range)	95.5 (46.0–167.0)	99.0 (34.0–171.0)	0.389
PLT, × 109/L			
median (range)	38.0 (2.0–292.0)	46.0 (2.0–354.0)	0.035
LDH (u/L)			
median (range)	1022.5 (181.0–8079.0)	339.5 (104.0–973.0)	
>1000 (%)	49.2	31.2	0.033
Blasts (%) median (range)			
bone marrow	90.4 (52.0–99.6)	84.4 (27.6–99.5)	0.006
peripheral blood	71.0 (0–99.0)	68.0 (0–97.0)	0.621
B or T lineage (%)			0.847
B-ALL	69.7	68.2	
T-ALL	30.3	31.8	
Immune phenotype, %			
CD34+	70.8	67.1	0.635
HLA-DR+	80.0	75.0	0.506
CD10+	71.9	75.0	0.691
CD19+	68.3	69.3	0.901
CD20+	39.3	40.0	0.935
CD22+	49.1	56.2	0.425
CD13+	39.1	44.0	0.556
CD33+	58.7	35.4	0.006
CD2+	25.9	25.4	0.945
CD3+	15.8	20.0	0.540
CD5+	27.5	32.8	0.529
CD7+	32.7	32.9	0.988

(*Continued*)

**Characteristics**	***c-MYC* high expression (*N* = 66)**	***c-MYC* low expression (*N* = 85)**	***P* value**
Extramedullary infiltration (%)			
liver	34.8	15.5	0.006
spleen	53.0	36.1	0.039
lymph node	54.5	48.2	0.441
Genetics (%)			
Complex karyotype	9.8	13.6	0.543
Complete remission rate (%)	82.3	95.8	0.010

Supplementary Table S2: The correlation of expression of *MYCBP2* with clinical features in acute lymphoblastic leukemia

Characteristics	*MYCBP2* high expression (*N* = 65)	*MYCBP2* low expression (*N* = 66)	*P* value
Age (years)			
median (range)	32 (14–75)	32.5 (14–77)	0.657
Sex (%)			
male	64.6	60.6	0.635
WBC, × 10^9/L			
median (range)	30.2 (0.9–183.9)	46.5 (2.0–626.0)	0.025
HGB, g/L			
median (range)	101.5(42.0–171.0)	97.0 (34.0–151.0)	0.782
PLT, × 109/L			
median (range)	41.5 (2.0–292.0)	42.0 (5.0–354.0)	0.779
LDH (u/L)			
median (range)	746.5 (124.0–8601.0)	1038.0 (131.0–8702.0)	
>1000 (%)	34.5	50.8	0.074
Blasts (%) median (range)			
bone marrow	87.2 (27.6–98.8)	88.8 (36.4–99.5)	0.042
peripheral blood	70.0 (0–97.0)	64.0 (0–99.0)	0.805
B or T lineage (%)			0.077
B-ALL	72.3	57.6	
T-ALL	27.7	42.4	
Immune phenotype, %			
CD34+	64.1	73.8	0.242
HLA-DR+	72.4	76.0	0.672
CD10+	74.1	73.2	0.911
CD19+	71.7	58.9	0.149
CD20+	41.1	43.1	0.829
CD22+	62.7	48.1	0.121
CD13+	33.9	50.0	0.073
CD33+	32.3	57.1	0.004
CD2+	30.6	28.3	0.798
CD3+	12.2	30.5	0.023
CD5+	29.8	40.4	0.271
CD7+	32.0	41.1	0.334

(*Continued*)

Characteristics	*MYCBP2* high expression (*N* = 65)	*MYCBP2* low expression (*N* = 66)	*P* value
Extramedullary infiltration (%)			
Liver	15.4	35.7	0.007
Spleen	35.9	54.3	0.033
Lymph node	49.2	56.5	0.398
Genetics (%)			
Complex karyotype	14.6	12.5	0.765
Complete remission rate (%)	92.7	85.5	0.213

Supplementary Table S3: The correlation of expression of *c-MYC*^{high}*MYCBP2*^{low}with clinical features in acute lymphoblastic leukemia

Characteristics	*c-MYC*^{high} *MYCBP2*^{low} (*N* = 20)	*c-MYC*^{low} *MYCBP2*^{high} (*N* = 31)	*P* value
Age (years)			
median (range)	40 (14–77)	30 (14–75)	0.344
Sex (%)			
male	70.0	64.5	0.685
WBC, × 10^9/L			
median (range)	101.5 (5.3–437.4)	29.4 (1.5–183.9)	0.007
HGB,g/L			
median (range)	76.0 (53.0–143.0)	99.0 (42.0–171.0)	0.244
PLT, × 109/L			
median (range)	16.5 (5.0–74.0)	39.0 (2.0–267.0)	0.013
LDH (u/L)			
median (range)	1635.0 (191.0–8079.0)	739.0 (124.0–8601.0)	0.076
>1000 (%)	70.0	29.0	0.004
Blasts (%) median (range)			
bone marrow	93.6 (65.2–98.6)	80.2 (27.6–97.2)	0.001
peripheral blood	83.5 (4.0–99.0)	70.5 (0–97.0)	0.513
Immune phenotype, %			
CD34+	90.0	61.3	0.025
HLA-DR+	90.9	69.2	0.229
CD10+	69.2	80.8	0.447
CD19+	53.3	74.1	0.172
CD20+	41.7	37.5	1.000
CD22+	41.7	65.4	0.289
CD13+	65.0	40.0	0.083
CD33+	80.0	25.8	0.000
CD2+	30.8	30.0	1.000
CD3+	47.1	23.8	0.178
CD5+	41.7	35.0	0.724
CD7+	42.9	33.3	0.724
Extramedullary infiltration (%)			
liver	75.0	19.4	0.000
spleen	75.0	33.3	0.004
lymph node	60.0	48.4	0.417
Genetics (%)			
Complex karyotype	7.1	14.3	0.635
Complete remission rate (%)	60.0	92.0	0.027

Supplementary Table S4: The correlation of expression of *Ikaros* with clinical features in acute lymphoblastic leukemia

Characteristics	*Ikaros* high expression (*N* = 79)	*Ikaros* low expression (*N* = 53)	*P* value
Age (years)			
median (range)	32 (14–75)	36 (14–77)	
Sex (%)			
male	57.0	64.2	0.409
WBC, × 10^9/L			
median (range)	29.5 (1.0–626.0)	64.9 (1.0–488.0)	0.016
HGB, g/L			
median (range)	100.0 (34.0–167.0)	95.0 (44.0–171.0)	0.752
PLT, × 109/L			
median (range)	48.0 (2.0–292.0)	41.0 (2.0–354.0)	0.114
LDH (u/L)			
median (range)	761.5 (124.0–8702.0)	985.0 (191.0–8079.0)	0.185
Blasts (%) median (range)			
bone marrow	86.2 (27.6–99.5)	88.0 (49.6–99.6)	0.459
peripheral blood	61.0 (0.0–10.0)	70.5 (0.0–99.0)	0.141
B or T lineage (%)			
B-ALL	78.5	60.4	
T-ALL	21.5	39.6	0.024
Immune phenotype, %			
CD34+	63.2	72.0	0.303
HLA-DR+	82.9	70.7	0.134
CD10+	70.4	80.4	0.226
CD19+	78.1	59.1	0.028
CD20+	44.8	34.9	0.303
CD22+	55.6	46.5	0.348
CD13+	33.3	57.1	0.009
CD33+	40.5	60.0	0.033
CD2+	21.5	25.0	0.682
CD3+	13.4	25.6	0.107
CD5+	23.4	41.0	0.059
CD7+	20.9	50.0	0.002
Extramedullary infiltration (%)			
Liver	11.4	41.5	0.000
Spleen	34.6	56.6	0.013
lymph node	41.8	57.7	0.074

(*Continued*)

Characteristics	*Ikaros* high expression (*N* = 79)	*Ikaros* low expression (*N* = 53)	*P* value
Genetics (%)			
Complex karyotype	7.4	17.1	0.257
Complete remission rate (%)	94.3	79.2	0.013
